# Does attachment anxiety mediate the persistence of anxiety and depressive symptoms from adolescence to early adulthood?

**DOI:** 10.1007/s00127-024-02737-8

**Published:** 2024-08-01

**Authors:** Julie A. Blake, Hannah J. Thomas, Anita M. Pelecanos, Jake M. Najman, James G. Scott

**Affiliations:** 1 QIMR Berghofer Medical Research Institute, Herston, Queensland Australia; 2 Child Health Research Centre, The University of Queensland, South Brisbane, Queensland Australia; 3 Queensland Centre for Mental Health Research, Wacol, Queensland Australia; 4 Centre for Children’s Health Research, Children’s Health Queensland, South Brisbane, Queensland Australia; 5 School of Public Health, The University of Queensland, Herston, Queensland Australia

**Keywords:** Inner-working models, Relationships, Emotion, YSR, YASR

## Abstract

**Objectives:**

Depression and anxiety often emerge in adolescence and persist into early adulthood. Developing a greater understanding of the factors that influence their persistence may inform psychological interventions. Their association with an insecure attachment style is well established although the mediating role of attachment anxiety in the persistence of depression and anxiety over time has not been examined. This study aimed to examine if anxious attachment mediated depression and anxiety from adolescence to early adulthood.

**Methods:**

Data from 3,436 participants in a longitudinal birth cohort study were examined. At 14-years and 21-years, participants completed the Achenbach Youth Self Report (YSR) and the Achenbach Young Adult Self-Report (YASR) respectively. At 21-years, participants completed the Attachment Style Questionnaire (ASQ). Attachment anxiety as a mediator for the persistence of anxiety/depressive symptoms from 14- to 21-years was examined.

**Results:**

Attachment anxiety accounted for approximately 60% of the persistence of anxiety and depressive symptoms at 14- and 21- years after adjusting for covariates. Results were similar when stratifying by males and females.

**Conclusions:**

Attachment anxiety significantly contributes to the persistence of anxiety and depressive symptoms from adolescence into early adulthood for both males and females. Incorporating interventions that address attachment anxiety in adolescents may improve the response to therapy for anxiety and depression.

**Supplementary Information:**

The online version contains supplementary material available at 10.1007/s00127-024-02737-8.

## Introduction

Anxiety and depression are highly prevalent and are the leading causes of disability in young people worldwide [1]. The incidence of anxiety and depression has significantly increased globally, with approximately one in four young people experiencing clinically elevated symptoms of depression, and one in five clinically elevated levels of anxiety [2, 3]. Symptoms of anxiety often first emerge in childhood and adolescence, depression commonly onsets in adolescence, and both sets of symptoms often persist into adulthood [4, 5]. Developing a greater understanding of the factors that influence their persistence may inform new treatments and/or enhance the existing effective treatments for anxiety and depressive symptoms. Attachment theory is a valuable framework through which to understand the development of inner working models of interpersonal relationships and their influence on mental health and illness. From early childhood, bonds with caregivers shape the way people think, feel, and behave in close relationships [6]. Healthy attachments with caregivers in childhood and adolescence support cognitive, social, and emotional development [7]. Disruptions to attachment bonds through inconsistent and unpredictable caregiving, child maltreatment or family conflict on the other hand, can lead to the formation of insecure attachment styles [8].

Anxious attachment is a type of insecure-attachment characterized by chronic insecurities around rejection and abandonment, and a preoccupation over whether an individual’s emotional needs will be met by others [9]. This style of attachment is frequently correlated with symptoms of depression and anxiety [10–13]. It is plausible that attachment anxiety could mediate the persistence of anxiety and depressive symptoms over time. Firstly, anxiously-attached individuals are more likely to engage in relationships marred by conflict and instability [14], in which they may be unable to access adequate support and connection to promote recovery from mental illness [15]. Secondly, a strong sense of self is demonstrated to be an essential element of recovery from mental illness [15], yet anxiously attached individuals exhibit an inner working model with a negative self-view [16]. Lastly, attachment anxiety can interfere with crucial emotional competencies (e.g., emotional intelligence and resilience) needed to effectively navigate stressful life events [17–19].

A widely used measure of attachment that considers inner-working models in young people is the Attachment Style Questionnaire (ASQ) [20]. Its dimensional approach to measuring attachment orientation offers an alternative over the use of mutually exclusive attachment categories that can miss within group variation and overlapping levels of attachment anxiety and avoidance [10, 21, 22]. The ASQ includes two domains reflecting anxious attachment, enabling a distinction between two fundamental facets underpinning attachment anxiety; ‘need for approval’ (NFA) and ‘preoccupation with relationships’ (PWR). These two dimensions of attachment-anxiety consistently, positively correlate with anxiety and depressive symptoms. For example, Colonnello et al. [23] found that higher need for approval and greater preoccupation with relationships were moderately associated with depressive symptoms in a cohort of 390 college students. The researchers considered emotion regulation as a mediator of this relationship and found only one component of emotion regulation (limited access to strategies) partially mediated the association. In another study, Riva Crugnola et al. [24] found the same two ASQ sub-scales were moderately to strongly associated with depression and anxiety in two groups of university students; one group who were receiving counselling (n = 370) and another group who were not receiving counselling (n = 318).

Despite a well-established relationship between attachment anxiety and depressive and anxiety symptomology and the persistent nature of these common mental health problems, no studies that examine attachment-anxiety as a mediator of depressive and anxiety symptoms over time could be identified to date. In their recent review, Fonagy et al. [25] highlight a need for greater understanding of the role that internal working models play in social functioning across the life course. Investigating key dimensions that underlie attachment anxiety may provide insights into the mechanisms responsible for the persistence of depressive and anxiety symptomology. This study aims to further the field by examining if attachment anxiety mediates the persistence of depressive and anxiety symptoms from early adolescence into early adulthood in an Australian community sample.

## Methods

### Sample

This study utilised data from the Mater-University of Queensland Study of Pregnancy (MUSP), a prospective birth cohort study of mothers and their offspring who received antenatal care at a tertiary Brisbane hospital between 1981 and 1984 (see Najman et al. [26, 27] for further details). Pregnant women attending their first prenatal appointment were consecutively invited to participate in the study. Participants provided informed written consent at each phase of data collection. Baseline data were collected on a total of 7,223 singleton, live-birth offspring and their 6,753 mothers. The discordance between the number of mothers and offspring arose because some mothers had more than one singleton offspring during the recruitment phase who were enrolled into the study. Mothers and offspring were followed prospectively when offspring were six-months, and five, 14 and 21 years of age. The 21-year follow-up occurred from 2003 to 2004. Ethical approval for this study was obtained from The University of Queensland Human Research Ethics Committee (mothers: B/555/SS/01/NHMRC − 30/11/2001, offspring: B/660/SS/01/NHMRC − 20/12/2001) and The Mater Human Research Ethics Committee (mothers: 505 A − 29/6/02, offspring: 506 A − 15/07/02). We excluded 3,787 (52.4%) offspring (and corresponding mothers) who were either lost to follow up (*n* = 3,680, 50.9%), or who had greater than 10% missing data on one or more of the three primary measures for this study (see below, *n* = 107, 1.5%), resulting in a final sample of 3,436 participating offspring.

### Instruments

At the 14-year follow-up, anxiety and depression symptoms were assessed using the Achenbach Youth Self Report anxiety/depression subscale (YSR-AD) [28, 29], a 13-item self-report subscale of a full 112-item questionnaire. The YSR-AD was designed to measure empirically derived internalizing symptoms, rather than two distinct diagnostic categories. The Youth Self Report is validated for children aged 11-18-years has shown adequate internal reliability in the study sample [30]. Participants respond to items such as “I feel lonely” based on the previous six-months on 3-point Likert scales (0 = rarely or never, 1 = sometimes and 2 = often). Items are combined to give an overall anxiety/depression score. The YSR-AD scale was treated as a continuous variable for the analyses.

At the 21-year follow-up, anxiety and depression symptoms were assessed using the Achenbach Young Adult Self-Report anxiety/depression subscale (YASR-AD) [31], a 17-item self-report subscale of a full 114-item questionnaire, which is very similar to the YSR-AD. The Young Adult Self Report is validated for individuals aged 18-30-years and has shown adequate internal reliability in the study sample [32]. Participants respond to items such as “I cry a lot” on 3-point Likert scales (0 = not true, 1 = somewhat true or sometimes true and 2 = very true or often true). Items are combined to give an overall anxiety/depression score. The YASR-AD scale was treated as a continuous variable for the analyses.

At the 21-year follow-up, offspring anxious attachment was assessed using the Attachment Style Questionnaire (ASQ) [20]. The ASQ is 40-item a self-report questionnaire measuring attachment orientation and inner-working models of self and others. Participants respond to items such as “I wonder why people would want to be involved with me” on 6- point Likert scales ranging from 1 = totally disagree to 6 = totally agree. A 33-item short form of the ASQ has been validated in the study sample [10]. The ASQ-33 includes two sub-scales comprising of 12 items, which assess attachment anxiety; the need for approval (NFA; related to fear of rejection), and preoccupation with relationships (PWR). These are correlated highly with depression and anxiety symptoms [10]. Items from the NFA and PWR subscales are combined to give an overall attachment anxiety score.

### Covariates

Data on of the following potential covariates were collected at 14-years (1994–1997): household income, maternal partner (birth father, new partner, no partner), openness of family communication (high, medium, low), problems in family communication (nil-few, some, many), maternal anxiety (yes, no), and maternal depression (yes, no). Sex at birth was also collected.

Maternal-reported household income at the 14-year follow-up was categorised as ≥/< $20,800AUD per annum, which distinguished between low and middle/high income households. Maternal depression and anxiety scores were measured using 14 items from the Delusions-Symptoms-States-Inventory anxiety and depression scale (DSSI-sAD) [33]; no anxiety or depression 0–3, anxiety/depression 4–7). Open communication and problem family communication were measured using maternal-reported items from the Parent-Adolescent Communication Scale (PACS) [34]; good < 33, fair 33–35, poor > 35).

### Statistical analysis

#### Missing data

A preliminary analysis of the data was performed to assess for attrition and missing data. Participants with less than 10% missing data on the anxiety/depression subscale of the YSR at 14-years and the YASR at 21-years, and the ASQ attachment anxiety subscale at 21-years, were retained. For each of these three subscales, Little’s Missing Completely At Random (MCAR) test was used to assess for the degree to which missing values appeared completely at random and were then imputed using a regression based simple imputation method.

A total 3,746 participants (51.9%) of the original study population completed at least one question on the ASQ (combined need for approval and preoccupation with relationships), at the 21-year follow-up. Of these, 33 (0.9%) had greater than 10% missing data (> 1 missing item). This process was replicated for the YSR and YASR anxiety/depression subscales, with a total of 63 (1.7%) participants and 32 (0.9%) removed from the total participants who answered at least one question at the 14- and 21- year follow-ups respectively. These data were identified as missing completely at random (YSR: χ^2^ (156) = 172, *p* = 0.174; YASR: χ^2^ (238) = 230, *p* = 0.632; ASQ: χ^2^ (132) = 141, *p* = 0.275) and were imputed using a simple regression-based imputation method whereby missing values are predicted using observed responses. A final sample of 3,436 (47.6%) participants completed > 90% of questions on all three primary measures (YSR anxiety/depression, YASR anxiety/depression and ASQ attachment anxiety).

Potential covariates were individually examined against each YASR anxiety/depression at 21-years, with those that were significantly correlated retained (*p* < 0.05). Secondly, the retained covariates were assessed for collinearity, with those producing a variance inflation factor (VIF) of < 5 [35] included in the final models. Multicollinearity was not detected between any of the covariates, resulting in a final set of seven confounders included in the mediation analyses.

### Mediation analysis

Data were analysed using R v4.1.1 [36]. The hypothesized mediation model (anxiety/depression at 14-years → attachment anxiety at 21-years → anxiety/depression at 21-years) was examined for the total sample, and separately for males and females, using the R package; *mediation* [37]. The indirect effects were tested using bootstrapping procedures. Unstandardized indirect effects were computed for each of 1,000 bootstrapped samples, and the 95% confidence interval was computed by determining the indirect effects at the 2.5th and 97.5th percentiles.

## Results

### Demographic characteristics of participants

Participant characteristics are presented in Table 1. At the 14-year follow-up, the majority of participants came from a middle-high income family (*n* = 2,730, 81.3%), with a maternal partner who was the same as at their birth (*n* = 2,408, 70.4%), had nil to few (maternally-reported) mother-child communication problems (*n* = 2,699, 78.7%) and whose mother did not experience clinically significant symptoms of anxiety (*n* = 2,815, 82.2%) or depression (*n* = 3,177, 92.7%). There were slightly more females (*n* = 1,816, 52.9%) than males (*n* = 1,620, 47.1%).

**Table 1:** Participant characteristics

	All participants *n* = 3,436
Sex at birth	
Male Female	1,620 (47.1) 1,816 (52.9)
YSR anxiety/depression 14-years	6.0 (5.0–8.0)
Maternal partner (*n* = 3,419)	
Different partner Same as at birth No partner	594 (17.4) 2,408 (70.4) 417 (12.2)
Maternal anxiety (*n* = 3,424)	
Not anxious Anxious	2,815 (82.2) 609 (17.8)
Maternal depression (*n* = 3,426)	
Not depressed Depressed	3,177 (92.7) 249 (7.3)
Family income (*n* = 3,359)	
Low Mid-high	629 (18.7) 2,730 (81.3)
Family communication (problems) (*n* = 3,429)	
Nil-few Some Many	2,699 (78.7) 415 (12.1) 315 (9.2)
Family communication (openness) (*n* = 3,429)	
Good Fair Poor	2,780 (81.1) 330 (9.6) 319 (9.3)
YASR anxiety/depression 21-years	7.0 (3.0–12.0)
ASQ attachment anxiety 21-years	37.0 (30.0–44.0)

Note. Not all participants had recorded information on all characteristics. Continuous variables expressed as median and interquartile range. Categorical variables expressed as numbers and column percentages. Not all column percentages total 100 due to rounding

**Table 2:** Direct and indirect effects of anxiety and depressive symptoms at 14-years on anxiety and depressive symptoms at 21-years

	Path a:	Path b:	Path c:	Path c’	Direct effect	Indirect effect	Proportion mediated
Unadjusted models							
Full sample	0.93^***^	0.43^***^	0.66^***^	0.26^***^	0.26 (0.21–0.32)	0.40 (0.35–0.45)	60%
Females	0.95^***^	0.45^***^	0.65^***^	0.22^***^	0.22 (0.15–0.30)	0.43 (0.36–0.49)	66%
Males	0.83^***^	0.40^***^	0.53^***^	0.20^***^	0.21 (0.18–0.30)	0.33 (0.25–0.41)	62%
Adjusted models							
Full sample	0.88^***^	0.42^***^	0.58^***^	0.21^***^	0.21 (0.16–0.27)	0.37 (0.32–0.42)	64%
Females	0.91^***^	0.45^***^	0.63^***^	0.22^***^	0.22 (0.14–0.30)	0.41 (0.34–0.48)	66%
Males	0.82^***^	0.39^***^	0.52^***^	0.20^***^	0.20 (0.10–0.29)	0.32 (0.24–0.41)	62%

Note. Path a: coefficient for anxiety/depression at 14-years on attachment anxiety at 21-years. Path b: coefficient for attachment anxiety at 21-years on anxiety/depression at 21-years. Path c: Total effect of anxiety/depression at 14-years and attachment anxiety at 21-years on anxiety/depression at 21-years. Path c’: direct effect of anxiety/depression at 14-years on anxiety depression at 21-years. Adjusted models accounted for maternal depression and anxiety, open and problem maternal adolescent communication, family income, maternal partner and sex at birth

### Mediation analysis

**Fig. 1:**
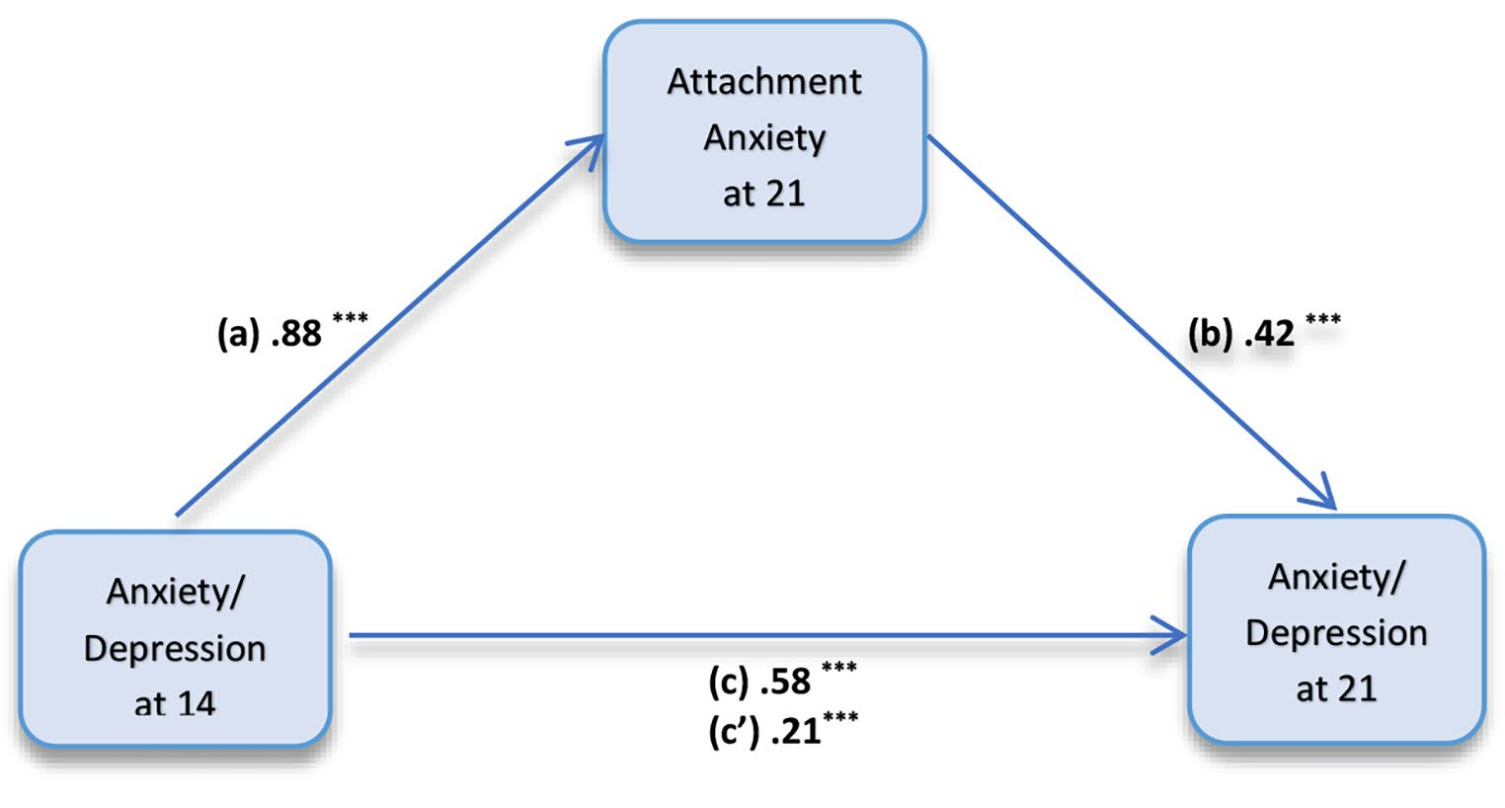
Mediation model; anxiety/depression at 14-years and anxiety/depression at 21-years with attachment anxiety at 21-years as the mediator. Model was adjusted for maternal depression and anxiety, open and problem maternal adolescent communication, family income, maternal partner and sex at birth

The effect of anxiety and depression at 14-years on anxiety and depression at 21-years was partially mediated via attachment anxiety at 21-years, both before and after the inclusion of covariates in the model (Table 2). For the adjusted model, the regression coefficient between YSR anxiety/depression (14 years) and YASR anxiety/depression (21 years), and the regression coefficient between attachment anxiety and YASR anxiety/depression (both at 21 years) were statistically significant. After adjustments, the bootstrapped unstandardized indirect effect was statistically significant (β *=* 0.37, 95% CI 0.32–0.42, *p* < 0.001), with 64% of proportion of the relationship between anxiety and depressive symptoms at age 14-years and 21-years mediated by attachment anxiety at age 21 (Fig. 1). For the sex stratified analysis, the bootstrapped unstandardized indirect effect was statistically significant after covariate adjustment for both males (β *=* 0.41, 95% CI 0.34–0.48, *p* < 0.001) and females (β *=* 0.32, 95% CI 0.24–0.41, *p* < 0.001), with 62% and 66% of proportion of the relationship between anxiety and depressive symptoms at 14- and 21-years mediated by attachment anxiety at age 21, respectively (Table 2).

A sensitivity analysis was conducted to test the mediating effects of the two attachment anxiety domains; need for approval (NFA) and preoccupation with relationships (PWR). Results were comparable to that of the main model, with NFA and PWR accounting 58% and 52% of the relationship between anxiety and depressive symptoms at age 14- and 21-years (β *=* 0.34 95% CI 0.29–0.39, *p* < 0.001; β *=* 0.30, 95% CI 0.25–0.35, *p* < 0.001, respectively). Results are shown in Supplementary Table 1.

## Discussion

The relationship between attachment anxiety and anxiety and depressive symptoms has been extensively studied [12, 13]. Given the persistent nature of anxiety and depression, the current study aimed to further extend previous findings by examining attachment anxiety in early adulthood as a potential mediator of anxiety and depressive symptoms from early adolescence to early adulthood. The results confirmed an indirect pathway via anxious attachment, suggesting that attachment anxiety contributes to persistent symptoms of anxiety and depression during the transitional years from adolescence into early adulthood. Attachment anxiety accounted for approximately 60% of the relationship between anxiety and depressive symptoms at 14- and 21-years after accounting for a range of environmental and familial confounding factors. The mediating effects were consistent across sexes and for two distinct attachment anxiety domains: need for approval and preoccupation with relationships, both exhibiting similar effect sizes in the overall model.

The findings of the current study strengthen insight into the role of attachment anxiety in the persistence of anxiety and depressive symptoms over time. The influences of attachment anxiety on anxiety and depressive symptoms throughout adolescence and adulthood can be understood through several possible explanations. Adolescents exhibiting anxiety and depressive symptoms are more likely to experience heightened insecurity within their parental attachment bonds [38, 39]. In turn, insecure attachment can predispose an individual not only to develop anxiety and depression, but for its persistence over time [40]. A study of over 1,700 young people aged 9 to 17 found for example, that attachment anxiety predicted increases in depressive symptoms two-years later, via reduced emotional regulation strategies [41]. Another study of 350 participants aged 11 to 17 found attachment anxiety predicted increases in anxious and depressive symptoms five-months later, even after controlling for baseline symptoms [42]. Adolescents with an anxious attachment style may adopt maladaptive coping strategies and develop a decreased sense of self-worth [43], poor self-esteem [42] and experience emotion regulation difficulties [23], all of which are correlated with depression [44, 45].

Interventions that address attachment anxiety in treating young people with anxiety and depression may improve mental health outcomes in young people. Secure attachment styles during childhood support adolescent psychological well-being and development [7]. Although previously thought to be stable, attachment styles can still be altered during adolescence, creating an opportunity for prevention and intervention, particularly in addressing common mental health symptoms prevalent among young people in western societies. Attachment-focused interventions in childhood take an interpersonal approach involving the carer and/or family and seek to restore the secure parent-child relationship that is required for the young person to develop adaptive skills needed to sustain psychological well-being [46, 47]. While attachment-based intervention frameworks for adolescent populations have been developed, they are currently underutilised [48].

Cognitive Behavioural Therapy (CBT) is the currently recommended first line treatment for anxiety and depression in adolescents [49]; however, not everyone responds positively, and approximately one in ten young people with anxiety, relapse [50]. In young people with depression, a meta-analysis found an increase in treatment effects of CBT when there was parental involvement [51]. The authors hypothesised that while not a treatment aim of CBT, an enhancement of attachment bonds may have led to the improved treatment outcomes. Standard therapeutic interventions that focus on strengthening interpersonal relationships may also offer an alternative treatment option for young people experiencing anxiety and depression. Incorporating interventions to address attachment anxiety in current treatments for adolescents might improve treatment response and reduce relapse. Interpersonal Therapy for Adolescents (IPT-A) for example, is a therapeutic approach that targets relational skills in adolescents with depression. IPT-A is shown to be effective in treating depression in adolescents [52] with recent evidence indicating IPT-A may also be effective at reducing concurrent attachment insecurity [53]. This finding further underscores the need to address attachment related difficulties when treating anxiety and depression in young people.

Research also suggests that better patient outcomes could be attained through the integration of an attachment framework into treatment models by involving caregivers and addressing attachment wounds [46, 54]. A clinical trial of an attachment-based program for parent-adolescent dyads (*n* = 540) found reductions in attachment anxiety were associated with clinically significant symptom reductions in internalizing symptoms [55]. Studies of attachment-based family therapy are also shown to be effective in reducing depressive symptoms and improving emotion regulation and interpersonal skills in adolescents [47]. Studies that incorporate longitudinal follow-up of such interventions are needed to demonstrate if improvements in attachment-focused treatments in adolescence are sustained and also to evaluate their effectiveness in preventing or reducing the anxiety and depression in early adulthood.

### Strengths and limitations

This study has a number of strengths. It uses a large community sample broadly representative of the general Australian population. The longitudinal and multi-generational nature of the cohort enabled the inclusion of important environmental and familial covariates into the analysis. Moreover, attachment orientation was measured using a dimensional, continuous measure reflecting broad inner working models of interpersonal relationships. We have previously demonstrated that attachment anxiety co-occurs with attachment avoidance which implies that attachment orientation lies on a security-insecurity continuum with individuals generally experiencing concordant levels of attachment anxiety and avoidance [10].

The results of this study; however, should be interpreted within the context of several limitations. The first limitation concerns the measures that were used. The YSR and YASR anxious/depressed syndrome scales are combined symptom scales and are not intended to be separated [56]. This inhibited the ability to examine potential differential mediating effects of attachment anxiety on anxiety and depression symptoms separately. Research shows that symptoms of anxiety and depression are highly co-morbid [5]. While it is possible to derive DSM oriented anxiety and depression subscales from the YSR and YASR, prior research in the study sample shows that these subscales perform poorly in discriminating between depression and anxiety as separate conditions [32]. Furthermore, self-report symptom measures rather than diagnostic interviews were used in this study. Mental health; however, exists on a continuum and the symptom measures reflect the real-world experience of mental health and illness.

The second limitation concerns temporal ordering assumptions of mediation analysis which ideally requires measures to be administered at three time-points in order to test causal mediation effects *between* the exposure and outcome. Mediation analyses are however frequently conducted using cross-sectional data, arguing that bias is minimised when measures are highly stable [57]. The current study leverages secondary data from a larger longitudinal study, that enabled a partially sequential mediation analysis across two time points spanning a seven-year period. While it is acknowledged that attachment style was measured at only one time point (21-years; concurrently with the outcome) research has demonstrated that attachment styles, while somewhat malleable in adolescence, tend to be stable by adulthood [8]. Therefore, attachment patterns in early adulthood reported in the current study were highly likely to persist during the study period. Third, approximately half of the study sample were lost to attrition at the 21-year follow-up. Previous research shows however, that differential attrition rarely affected the estimates of association [58].

## Conclusions

The findings of this study contribute to a better understanding of the mechanism by which anxiety and depressive symptoms may persist from adolescence into early adulthood. Attachment anxiety in early adulthood accounted for over half of the relationship between anxiety and depressive symptoms in early adolescence with early adulthood after accounting for a wide range of familial and environmental factors. While the results of this study demonstrate the importance of attachment styles in the persistence of mental health problems from adolescence to early adulthood, further studies that incorporate measures of attachment and anxiety and depressive symptoms across a greater number of time points would provide greater support for causal inference implied by mediation models. In conclusion, this highlights the potential role of incorporating psychological interventions that specifically address attachment anxiety in the treatment of adolescents with common mental health problems to reduce illness persistence into adulthood.

## Electronic supplementary material

Below is the link to the electronic supplementary material.

Supplementary Material 1
