# Supplementary material for: Does attachment anxiety mediate the persistence of anxiety and depressive symptoms from adolescence to early adulthood?
Source: Soc Psychiatry Psychiatr Epidemiol. 2024 Aug 1;60(2):453–61. doi: 10.1007/s00127-024-02737-8 (PMC11839844; doi:10.1007/s00127-024-02737-8)
Supplement: Supplementary file 1 — Supplementary Material 1 [file 127_2024_2737_MOESM1_ESM.docx]

**Supplementary materials**

**Supplementary Table 1. Direct and indirect effects of anxiety and depressive symptoms at 14-years on anxiety and depressive symptoms at 21-years.**

| Mediator | Path a: | Path b: | Path c: | Path c’ | Direct effect | Indirect effect | Proportion mediated |
| --- | --- | --- | --- | --- | --- | --- | --- |
| Need for approval | .84^***^ | .34^***^ | .58^***^ | .25^***^ | 0.25 (0.19 – 0.31) | 0.34 (0.29 – 0.39) | 58% |
| Preoccupation with relationships | .64^***^ | .30^***^ | .58^***^ | .28^***^ | 0.28 (0.22 – 0.35) | 0.30 (0.25 – 0.35) | 52% |

Note. Path a: coefficient for anxiety/depression at 14-years on attachment anxiety at 21-years. Path b: coefficient for attachment anxiety at 21-years on anxiety/depression at 21-years. Path c: Total effect of anxiety/depression at 14-years and attachment anxiety at 21-years on anxiety/depression at 21-years. Path c’: direct effect of anxiety/depression at 14-years on anxiety depression at 21-years. Adjusted models accounted for maternal depression and anxiety, open and problem maternal adolescent communication, family income, maternal partner and sex at birth
